# Rapid complete remission after one cycle of isatuximab-based quadruplet regimen in 1q21-positive primary plasma cell leukemia: a case report

**DOI:** 10.3389/fimmu.2026.1815111

**Published:** 2026-05-08

**Authors:** Lei Pang, Minran Zhou, Chunyan Chen, Ping Qin, Shuxin Yan, Xiaoya Dong, Ran Wang

**Affiliations:** 1 Department of Hematology, Qilu Hospital of Shandong University, Jinan, China; 2 Shandong Key Laboratory of Hematological Diseases and Immune Microenvironment, Jinan, China; 3 Shandong Provincial Clinical Research Center for Hematological Diseases, Jinan, China

**Keywords:** 1q21 gain, case report, CD38 monoclonal antibody, isatuximab, novel therapy, primary plasma cell leukemia

## Abstract

Primary plasma cell leukemia (pPCL) is a rare and highly aggressive plasma cell malignancy. Patients often present with a large number of circulating plasma cells and extramedullary organ involvement at an early stage of the disease, frequently accompanied by severe infections and organ dysfunction, resulting in a poor overall prognosis. Current treatment strategies for pPCL are largely extrapolated from intensive induction regimens used for multiple myeloma; however, therapeutic outcomes remain limited, particularly in patients with high-risk cytogenetic abnormalities and impaired immune function. Isatuximab, an anti-CD38 monoclonal antibody, has demonstrated favorable efficacy in patients with relapsed/refractory multiple myeloma, but clinical evidence supporting its use in pPCL is still lacking. Here, we report a critically ill, newly diagnosed patient with primary plasma cell leukemia harboring 1q21 gain and extensive malignant plasma cell infiltration in the pleural effusion, who achieved complete remission (CR) after one cycle of an isatuximab-based combination regimen.

## Introduction

Plasma cell leukemia is the rarest and most aggressive subtype of plasma cell dyscrasias, characterized by the presence of a large number of monoclonal plasma cells in the peripheral circulation and frequent extramedullary involvement. According to the 2021 revised criteria of the International Myeloma Working Group (IMWG), after fulfilling the diagnostic criteria for multiple myeloma, primary plasma cell leukemia (pPCL) is defined by the presence of ≥5% circulating plasma cells in peripheral blood or an absolute plasma cell count ≥2 × 10^9^/L. In contrast, leukemic transformation occurring in patients with pre-existing multiple myeloma is defined as secondary plasma cell leukemia. Primary plasma cell leukemia has an abrupt onset, rapid progression, and high tumor burden, with prominent clinical manifestations. Patients commonly present with severe anemia, thrombocytopenia, renal dysfunction, and serious infections, and face a high risk of early mortality.

All cases of pPCL should be considered ultra-high-risk plasma cell malignancies. Previous studies have reported a median overall survival of approximately 13 months (1, 2). However, due to its extremely low incidence, it is difficult to collect sufficient cases to conduct comprehensive studies, and robust evidence to establish optimal treatment strategies is lacking. Current therapeutic approaches for pPCL are mainly derived from intensified induction regimens used in multiple myeloma. For eligible patients, combination regimens based on four agents—including an anti-CD38 monoclonal antibody, a proteasome inhibitor, an immunomodulatory drug, and dexamethasone—are recommended, followed by sequential autologous stem cell transplantation (ASCT) and consolidation or maintenance therapy when feasible. During induction therapy, not only rapid reduction of tumor burden but also achievement of deep remission is crucial to facilitate subsequent transplantation and long-term disease control. Nevertheless, even in the era of novel agents, long-term survival of pPCL patients has not been fundamentally improved, particularly in those with high-risk cytogenetic abnormalities such as 1q21 gain and del(17p), who continue to have a poor prognosis (6). Therefore, exploring treatment strategies with rapid onset of action and favorable tolerability in critically ill, high-risk pPCL patients is of great clinical significance.

Isatuximab is a monoclonal antibody targeting CD38 and has shown favorable efficacy in patients with relapsed/refractory multiple myeloma (3, 4); however, reports on its application in pPCL are scarce. A previous report described two patients with secondary plasma cell leukemia who were treated with elotuzumab- or isatuximab-based combination regimens (EPd or Isa-Pd). One patient receiving Isa-Pd achieved stringent complete remission after two cycles and maintained a sustained response during follow-up. Although these observations suggest that isatuximab may have activity in aggressive plasma cell disorders, clinical evidence supporting its use in newly diagnosed primary plasma cell leukemia remains extremely limited. In this study, we report a critically ill patient with pPCL harboring high-risk 1q21 gain who achieved complete remission (CR) after one cycle of an isatuximab-based four-drug combination regimen, with rapid improvement in clinical symptoms. This regimen not only rapidly reduced the high tumor burden and induced CR in this high-risk, critically ill patient, but also laid a foundation for subsequent stem cell transplantation and long-term disease control, providing clinical evidence for the treatment of pPCL.

## Case presentation

**Figure 1:**
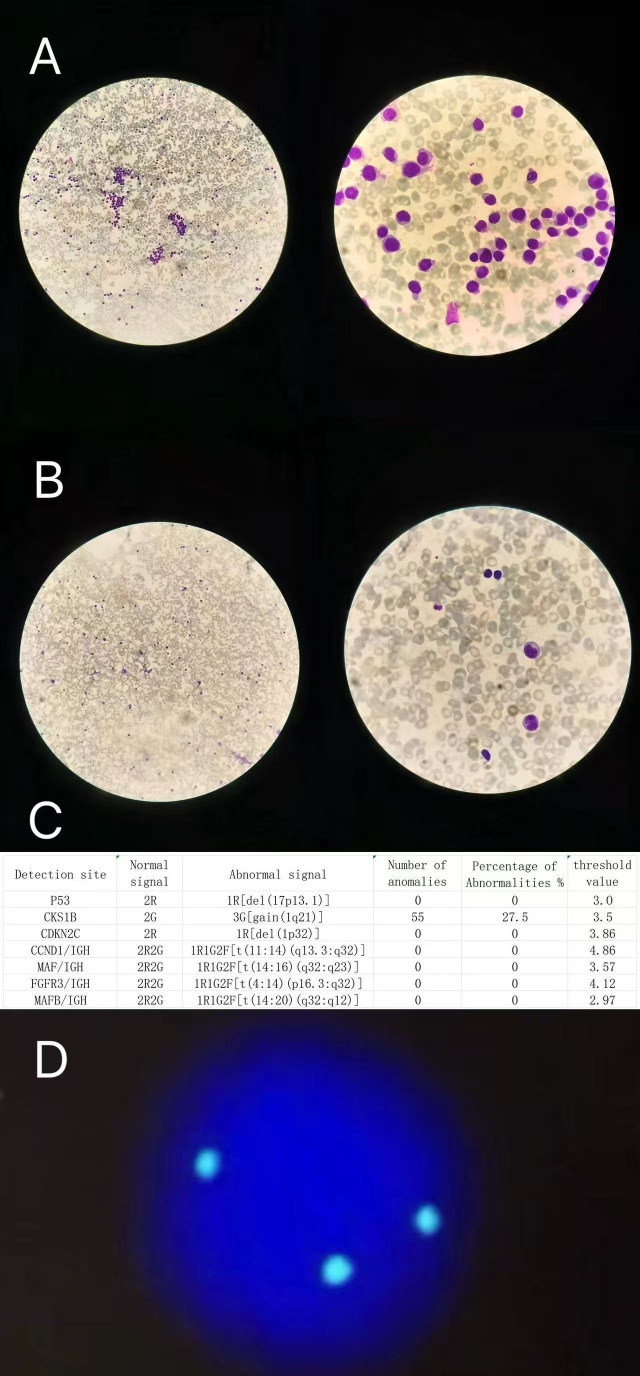
Bone marrow cytology before and after treatment, and baseline fluorescence *in situ* hybridization (FISH) findings. **(A)** Bone marrow smear at diagnosis showing extensive infiltration of immature plasma cells (left column ×100, right column ×1000). **(B)** Bone marrow after one treatment cycle showing marked reduction of plasma cells and recovery of hematopoiesis (left column ×100, right column ×1000). **(C)** Bone marrow FISH results. **(D)** FISH image of CKS1B (3G, 1q21 gain).

A 69-year-old female was admitted to our hospital with complaints of dyspnea and purpura of the right upper limb for one month. The patient had no significant past medical history and no known chronic comorbidities prior to admission. On admission, the patient exhibited significant bleeding tendency and fatigue, with a weight loss of 20 kg over the preceding three months. Complete blood count revealed a white blood cell count of 11.45 × 10^9^/L, hemoglobin of 93 g/L, and platelet count of 19 × 10^9^/L. Biochemical tests showed uric acid 566 μmol/L, lactate dehydrogenase 263 IU/L, β_2_-microglobulin 11.1 mg/L, creatinine 84 μmol/L, calcium 2.31 mmol/L, albumin 41.5 g/L, globulin 15.1 g/L and NT-proBNP 3712 pg/mL. Quantitative immunoglobulin levels were markedly decreased, and serum immunofixation electrophoresis did not detect monoclonal immunoglobulin. Baseline urine protein electrophoresis showed no detectable monoclonal protein (M protein 0 g/24 h), and total 24-hour urinary protein excretion was within the normal range. Serum free light chain analysis demonstrated a markedly elevated κ light chain level of 252.32 mg/L and a suppressed λ light chain level of 6.23 mg/L, with an abnormal κ/λ ratio of 40.50. Bone marrow aspiration revealed hypercellular marrow with approximately 85% immature plasma cells, with marked suppression of granulocytic, erythroid, and megakaryocytic lineages. Peripheral blood smear showed immature plasma cells accounting for approximately 53% of leukocytes (**Figure 1A**). Flow cytometric analysis of bone marrow demonstrated abnormal plasma cells comprising 45.41% of nucleated cells, expressing CD38, CD138, CD56, BCMA, and cytoplasmic κ light chain, while lacking expression of CD19, CD45, and cytoplasmic λ light chain. Cytogenetic analysis revealed 1q21 gain, with FISH analysis demonstrating positivity in 55 out of 200 analyzed cells (27.5%) (**Figures 1C, D**). Fluorescence *in situ* hybridization (FISH) analysis did not detect other high-risk cytogenetic abnormalities, including del(17p), t(4;14), or t(14;16). Molecular analysis showed no detectable TP53 mutation, and IGH gene rearrangement testing was negative. Positron emission tomography/computed tomography (PET−CT) revealed multiple osteolytic lesions with elevated FDG uptake, bilateral pleural effusion, pelvic effusion, and inflammatory changes in both lungs; no other abnormal findings were identified within the scanned field. Given the absence of a prior history of multiple myeloma and in accordance with the revised IMWG diagnostic criteria, the patient was diagnosed with kappa light chain–only primary plasma cell leukemia.

After admission, the patient initially received supportive treatments including platelet transfusion, uric acid–lowering therapy, and empirical anti-infective therapy. Microbiological testing, including sputum cultures, did not identify bacterial or fungal pathogens. Given the presence of pneumonia and the patient’s profound immunocompromised status, broad-spectrum antimicrobial coverage was initiated to address potential occult or mixed infections. Comprehensive assessment revealed multiple adverse factors at diagnosis, including high tumor burden, significant bleeding tendency, pulmonary infection, and cardiac and renal dysfunction. To achieve rapid disease control and symptom relief, an Isa-VRD regimen (isatuximab, bortezomib, lenalidomide, and dexamethasone) was initiated on the basis of adequate supportive care. The treatment regimen consisted of intravenous isatuximab at 10 mg/kg (approximately 650 mg for this 65 kg patient), administered on days 1, 8, 15, 22, and 29. Dexamethasone was administered intravenously at a total dose of 40 mg twice weekly. Given the patient’s baseline condition, including active pulmonary infection and cardiac and renal insufficiency, dose adjustments were made for bortezomib and lenalidomide to improve tolerability. Specifically, bortezomib was administered subcutaneously at 1 mg/m² (approximately 1.6 mg per dose) on days 1, 8, 15, and 22, while lenalidomide was given at 10 mg once daily for 21 days followed by 7 days off. One treatment cycle was defined as 42 days.

**Figure 2:**
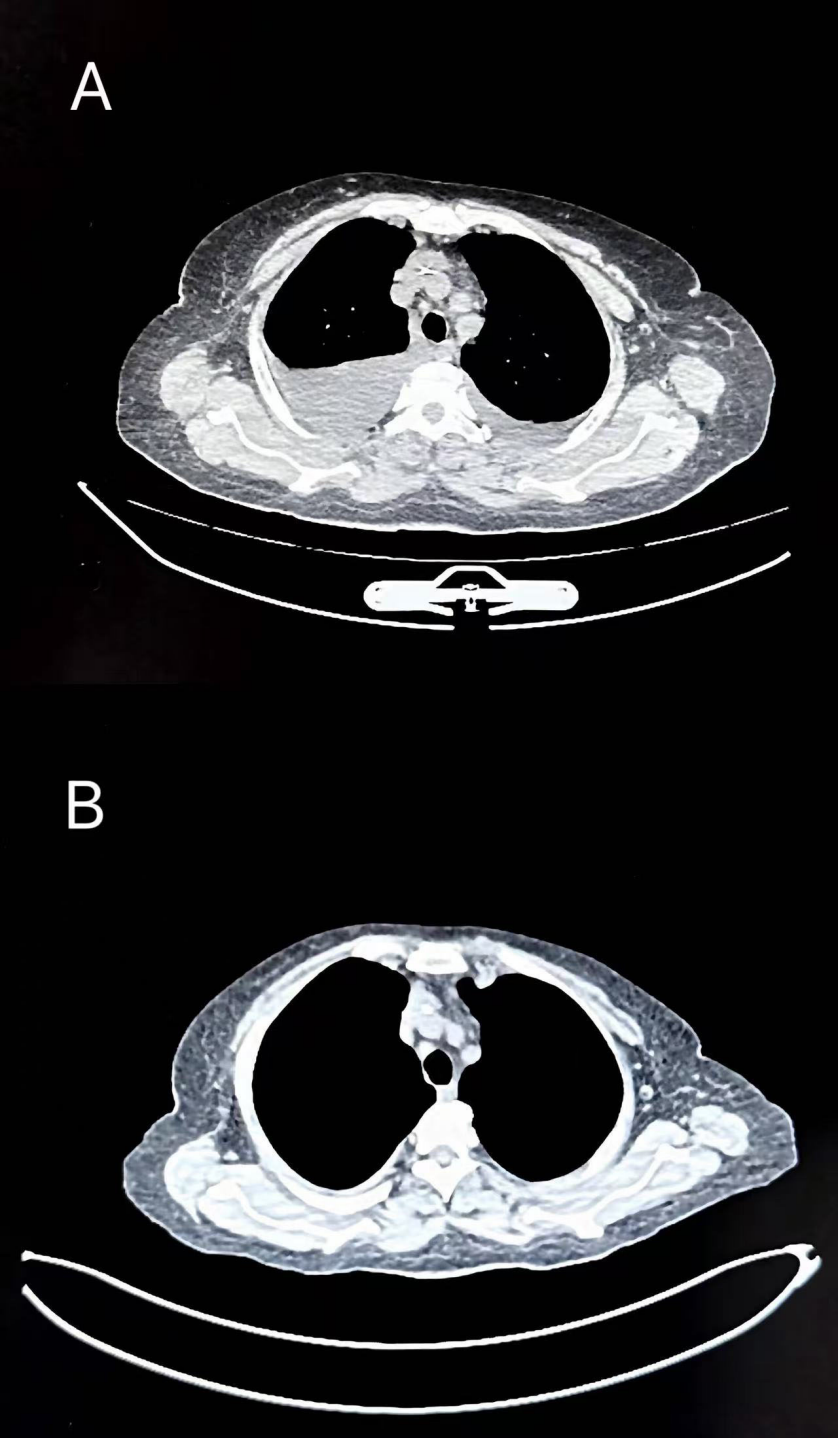
Chest CT findings. **(A)** Massive bilateral pleural effusion at diagnosis. **(B)** Near-complete resolution of pleural effusion (Chest CT performed on Day 32).

**Figure 3:**
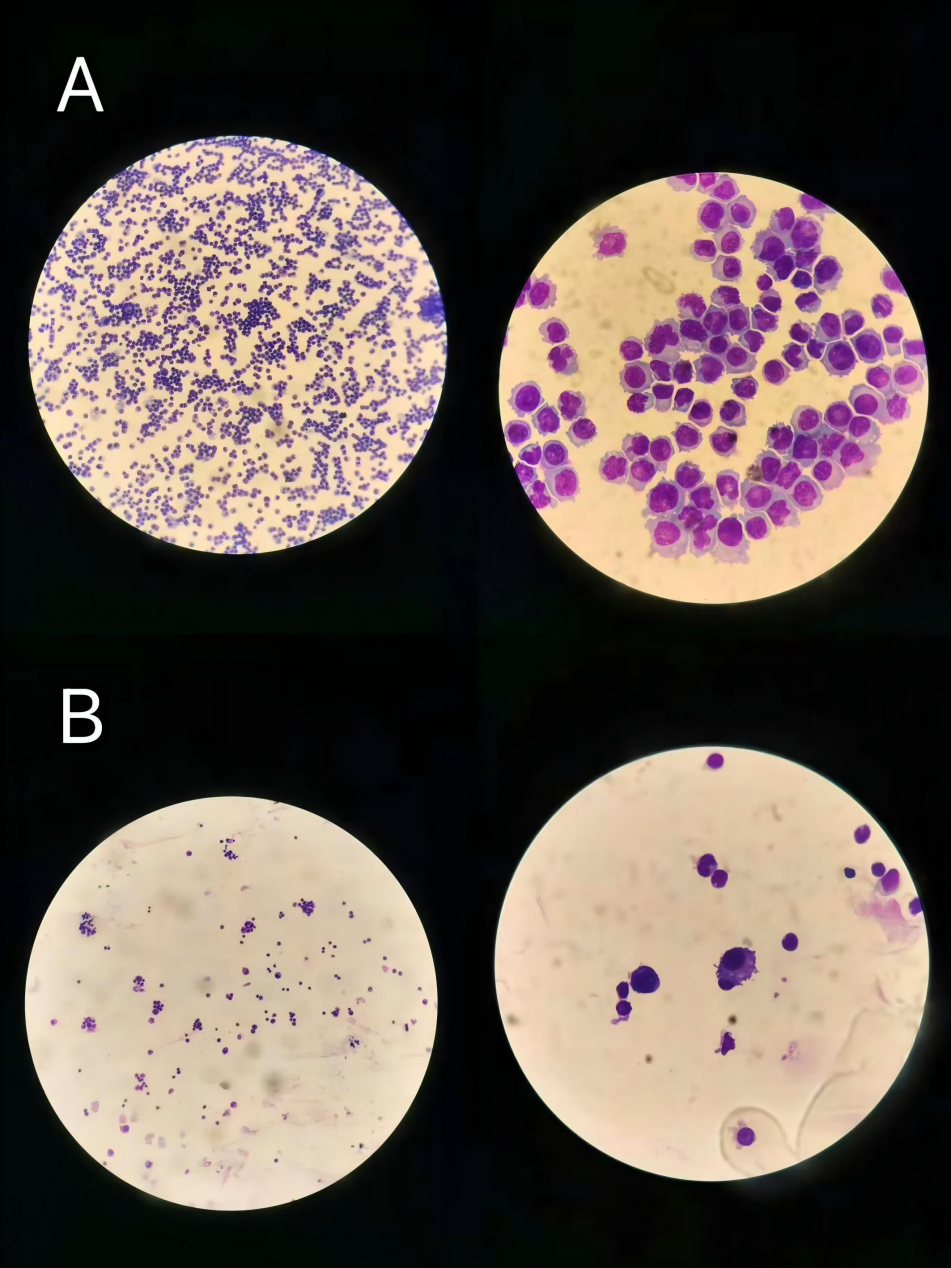
Cytological examination of pleural effusion before and after treatment. **(A)** Pleural effusion before treatment demonstrating numerous malignant plasma cells (left column ×100, right column ×1000). **(B)** Pleural effusion after treatment showing significant decrease in plasma cells (left column ×100, right column ×1000).

**Figure 4:**
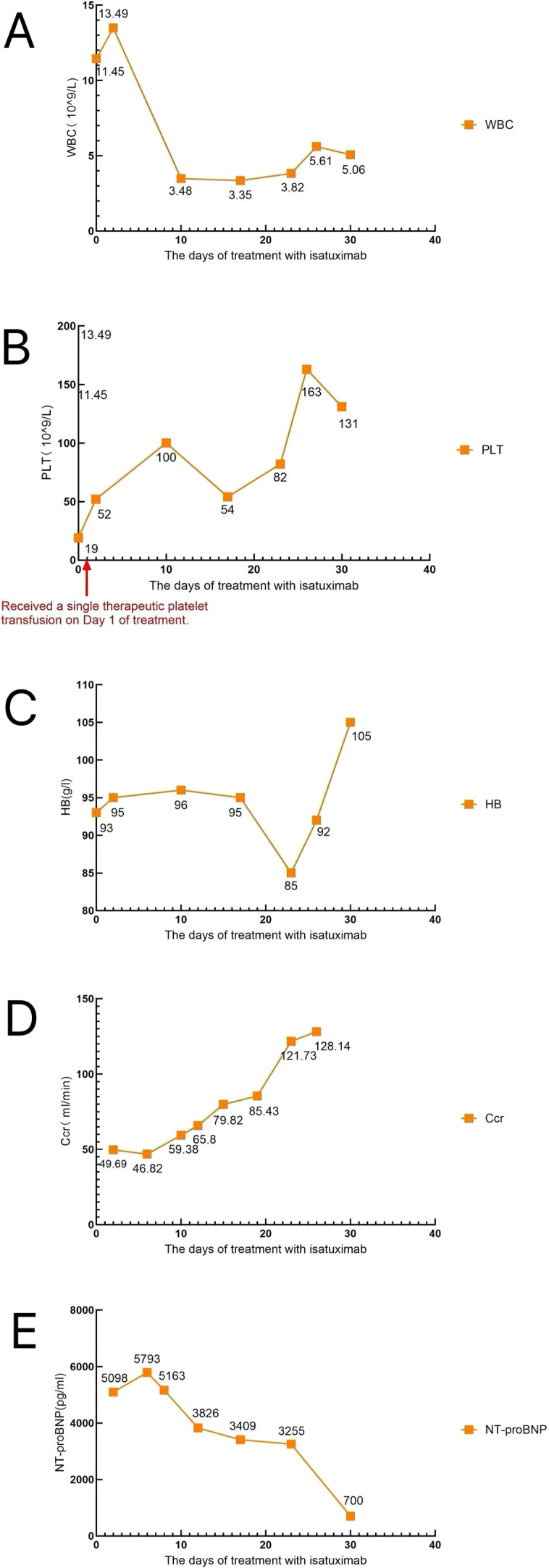
Dynamic changes in laboratory parameters during treatment. **(A)** White blood cell count. **(B)** Platelet count. **(C)** Hemoglobin level. **(D)** Creatinine clearance level. **(E)** NT-proBNP level.

During treatment, the patient developed atrial fibrillation and heart failure, and received comprehensive management including continued anti-infective therapy, correction of electrolyte disturbances, hepatoprotective and renoprotective measures, correction of hypoalbuminemia, parenteral nutritional support, component blood transfusion, optimization of cardiac function, and restoration of sinus rhythm. Following initiation of the Isa-VRD regimen, the patient’s clinical symptoms and laboratory parameters improved rapidly. On day 6 of treatment, chest CT demonstrated inflammatory changes in both lungs and massive bilateral pleural effusion, accompanied by incomplete expansion of the adjacent lung tissue (**Figure 2A**). Thoracentesis with catheter drainage was subsequently performed, yielding 800 mL of hemorrhagic pleural effusion fluid at the initial drainage. Cytological examination of the pleural fluid revealed a large number of immature plasma cells (**Figure 3A**). Flow cytometry demonstrated abnormal plasma cells accounting for 44.55% of nucleated cells, expressing CD38, CD138, CD56, CD117, and cytoplasmic κ light chain, while lacking CD19, CD45, and cytoplasmic λ light chain. No bacteria or fungi were observed on smear, and targeted sequencing for infectious pathogens in the pleural effusion was negative, suggesting extramedullary involvement. On day 10 (after the second dose of isatuximab), repeat blood tests showed a decrease in white blood cell count to 3.48 × 10^9^/L and an increase in platelet count to 100 × 10^9^/L (**Figures 4A, B**), following only a single platelet transfusion administered on day 1, suggesting that the sustained platelet recovery was more likely associated with treatment response rather than transfusion support alone. Lactate dehydrogenase levels rapidly declined to 197 IU/L, and the pre-existing bleeding tendency was controlled. With ongoing treatment and continuous pleural drainage, the volume of pleural effusion gradually decreased, and the color changed from bloody to yellow. On day 20 of treatment, repeat cytological examination of pleural effusion showed predominantly lymphocytes, with scattered small clusters of immature plasma cells, markedly reduced compared with the previous specimen (**Figure 3B**), indicating significant resolution of extramedullary disease involvement. On day 32, follow-up chest CT revealed no significant residual pleural effusion. The chest drainage tube was therefore removed, with a total drainage volume exceeding 10,000 mL (**Figure 2B**). Notably, these improvements occurred while the patient was still in the phase of infection control and bone marrow suppression, suggesting that tumor burden reduction preceded full hematologic recovery.

After one treatment cycle, the patient’s symptoms significantly improved. On day 30, repeat complete blood count was nearly normal, with white blood cell count of 5.06 × 10^9^/L, hemoglobin 105 g/L (**Figure 4C**), and platelet count 131 × 10^9^/L. NT-proBNP decreased to 700 pg/mL, and cardiac and renal functions were corrected (**Figures 4D, E**). Repeat serum free light chain analysis demonstrated κ light chain 4.01 mg/L, λ light chain 3.13 mg/L, with a κ/λ ratio of 1.2812. Serum and urine immunofixation electrophoresis did not detect any monoclonal immunoglobulin. Repeat bone marrow examination revealed active hematopoiesis with only 3% immature plasma cells and generally normal morphology and lineage ratios. No immature plasma cells were observed in the peripheral blood (**Figure 1B**). Bone marrow flow cytometry showed abnormal plasma cells accounting for only 0.08% of nucleated cells. According to the International Myeloma Working Group (IMWG) response criteria, the patient fulfilled the criteria for complete remission (CR), as evidenced by negative serum and urine immunofixation, normalization of the serum free light chain ratio, <5% plasma cells in the bone marrow, and disappearance of circulating plasma cells in the peripheral blood. This demonstrates that isatuximab combined with the VRD regimen can induce rapid and deep remission in pPCL with favorable safety.

## Discussion

Primary plasma cell leukemia (pPCL) is a rare plasma cell malignancy with extremely aggressive behavior, characterized by rapid disease progression and recognized as one of the plasma cell disorders with the poorest prognosis. Compared with multiple myeloma, pPCL is associated with a higher incidence of extramedullary infiltration, involving organs such as the liver, spleen, lymph nodes, serous cavities, and the central nervous system (5, 6). Clinically, patients exhibit biological features overlapping those of multiple myeloma and acute leukemia, and are frequently complicated by infection, bone marrow suppression, and end-organ failure, which markedly increases treatment difficulty and contributes to poor outcomes. Therefore, current guidelines emphasize that induction therapy should not only achieve rapid control of high tumor burden, but also strive for deep remission in order to establish a foundation for subsequent transplantation and long-term disease control. Despite the adoption of intensified multiple myeloma–based regimens and the development of novel therapeutic approaches specifically for PCL, overall survival in pPCL patients has improved only marginally, and prognosis remains unfavorable. In particular, for patients who are already in a critical condition at diagnosis, achieving effective disease control within a short period remains a major therapeutic challenge.

In the present case, the proportion of abnormal plasma cells in the peripheral blood was markedly elevated, bone marrow plasma cell infiltration exceeded 80%, and abnormal plasma cell infiltration with an immunophenotype identical to that of bone marrow plasma cells was detected in the pleural effusion, clearly indicating extramedullary involvement. Meanwhile, the patient was complicated by severe bleeding tendency, infection, renal impairment, and cardiac dysfunction, and harbored the high-risk cytogenetic abnormality of 1q21 gain. These features collectively suggested a critical disease status and poor prognosis. Previous studies have shown that conventional induction therapy often fails to achieve satisfactory outcomes in such patients and that intensive treatment regimens are poorly tolerated (2). Therefore, the central therapeutic challenge in this case was how to reduce tumor burden as safely and rapidly as possible and promptly improve clinical symptoms in the setting of poor overall condition.

CD38 is expressed at relatively low levels on normal bone marrow cells and lymphocytes, but is typically highly expressed on abnormal plasma cells in pPCL, making it a relatively stable and accessible therapeutic target (3, 4). The use of CD38 monoclonal antibody–containing regimens, particularly daratumumab-based therapy, has been shown to confer survival benefits in the treatment of primary plasma cell leukemia, with generally manageable tolerability (2). In this case, a CD38 monoclonal antibody–based four-drug combination regimen was selected with the aim of maximizing disease control during induction without significantly increasing treatment-related toxicity. The treatment decision highlights the importance of “clear targeting and rapid onset of action” in the management of critically ill patients with pPCL.

Daratumumab and isatuximab are currently approved CD38 monoclonal antibodies for the treatment of multiple myeloma. In the overall patient population, the two agents demonstrate comparable efficacy and are recommended in parallel as CD38-targeted therapeutic options in multiple clinical guidelines (10). However, in highly aggressive disease states characterized by severe immune dysfunction and extremely high tumor burden, differences in mechanisms of action may result in differential effects on early disease control. Compared with daratumumab, isatuximab not only mediates antibody-dependent cellular cytotoxicity (ADCC) and antibody-dependent cellular phagocytosis (ADCP), but can also directly induce apoptosis of CD38-positive tumor cells. In addition, the binding affinity of isatuximab is approximately 19-fold higher than that of other CD38 monoclonal antibodies, and its stronger inhibition of extracellular enzymatic activity may partially reverse adenosine-mediated immunosuppression within the tumor microenvironment, resulting in higher biological activity under comparable conditions (7). These mechanistic characteristics suggest potential advantages in disease states with high tumor burden and impaired immune effector cell function. Previous studies have demonstrated that in patients with relapsed or refractory multiple myeloma who are refractory to daratumumab, isatuximab combined with pomalidomide and dexamethasone can still achieve an overall response rate exceeding 50%, indicating that isatuximab may retain antitumor activity even in the setting of CD38 monoclonal antibody resistance (7, 11). Moreover, intensified combination regimens containing isatuximab (such as Isa-KRd and Isa-VRd) have been shown to induce a high proportion of deep responses and minimal residual disease (MRD) negativity in high-risk or transplant-ineligible patients. These mechanistic and clinical characteristics help explain why, in this case, despite concurrent infection and bone marrow suppression—conditions theoretically unfavorable for immune-dependent mechanisms—rapid tumor burden reduction was still observed following treatment with an isatuximab-containing four-drug regimen. Taken together, given comparable overall efficacy, the differentiated mechanisms of action of isatuximab, its documented activity after daratumumab resistance, and its ability to induce deep remission in high-risk patient populations support its biologically rational role as an alternative therapeutic option for plasma cell leukemia.

Extramedullary involvement is an important feature of pPCL and is closely associated with abnormalities in tumor cell adhesion molecules and enhanced migratory capacity (5). Mutations in genes encoding certain adhesion molecules (such as CD56, ICAM, NCAM, and LFA) can reduce the adhesion of malignant plasma cells to the bone marrow microenvironment, facilitating their entry into the peripheral circulation and subsequent extramedullary dissemination. In the present case, not only were large numbers of abnormal plasma cells detected in the peripheral blood, but abnormal plasma cell infiltration with an immunophenotype highly consistent with bone marrow plasma cells was also identified in the pleural effusion, indicating definite serous cavity involvement. Following isatuximab-based therapy, abnormal plasma cells in both the peripheral blood and pleural effusion declined synchronously and rapidly, and were essentially eliminated after one treatment cycle. This synchronous response suggests that isatuximab exerts strong disease control over both circulating and extramedullary plasma cells.

The patient also harbored a 1q21 gain. The 1q21 cytogenetic abnormality is associated with shortened overall survival in multiple myeloma and is recognized as an independent adverse prognostic factor. Many genes located at the 1q21 locus have been shown to be upregulated in aggressive plasma cell diseases, contributing to early disease progression, treatment resistance, and poor prognosis (6, 8). Subgroup analyses in multiple myeloma have demonstrated that patients with 1q21 abnormality have inferior survival compared with those without high-risk cytogenetic abnormalities, and that conventional proteasome inhibitors and immunomodulatory drugs show limited efficacy in this population. This high-risk genetic alteration significantly shortens progression-free survival (PFS) and overall survival (OS). Although autologous stem cell transplantation (ASCT) can improve survival outcomes in patients with 1q21 abnormality, it does not eliminate the adverse prognostic impact, and higher copy numbers are associated with poorer post-transplant OS (8, 11). With the expanding clinical application of CD38 monoclonal antibodies, patients with higher risk of disease progression associated with 1q21 abnormalities—including isolated 1q21 gain and amplification—have demonstrated PFS benefit and deeper responses when treated with isatuximab-containing combination regimens. These findings support isatuximab as an effective therapeutic option for patients with 1q21-positive pPCL (7). In this reported case, the pPCL patient with 1q21 gain achieved complete bone marrow remission after one cycle of isatuximab-based induction therapy, suggesting that this regimen may retain strong early antitumor activity even in the context of high-risk cytogenetic abnormalities.

In terms of safety, isatuximab is generally well tolerated, with no novel safety signals distinct from those observed with other anti-CD38 monoclonal antibodies. Its adverse event profile is relatively stable and predictable, and most events can be managed through early preventive monitoring and supportive care (4, 9). Hematologic toxicity is the most common adverse event associated with isatuximab therapy and can be mitigated by close monitoring and growth factor support, thereby minimizing its impact on treatment of the primary disease. Infusion-related reactions represent another common adverse event, most frequently occurring during the first administration. In addition, the immunosuppressive effects of isatuximab, particularly treatment-induced hypogammaglobulinemia, may increase the risk of infection. Overall, the adverse event profile of isatuximab is highly similar to that of daratumumab, and the safety characteristics of both agents are generally consistent across different combination regimens. In the present case, the patient successfully completed induction therapy under close supportive management without uncontrollable toxicities, indicating that the isatuximab plus VRD regimen is a feasible and safe option for critically ill patients with pPCL.

## Conclusion

Primary plasma cell leukemia is an extremely aggressive plasma cell malignancy, and patients often present with a massive tumor burden at the time of diagnosis. With the progressive clarification of the clinical characteristics of pPCL and the emergence of an increasing number of novel therapeutic agents, treatment options for pPCL have expanded; however, overall prognosis has improved only marginally. This report describes a critically ill patient with primary plasma cell leukemia who was treated with an isatuximab-based multidrug combination regimen. After one cycle of isatuximab plus VRD therapy, the patient achieved rapid and significant hematologic remission with favorable safety. After initial management at our center and clinical stabilization, the patient continued treatment at a local hospital and remains in remission pending autologous hematopoietic stem cell transplantation (ASCT). This case suggests that isatuximab-containing regimens may offer a potential advantage in rapidly reducing tumor burden in patients with primary plasma cell leukemia complicated by impaired immune function and prominent extramedullary involvement. Although conclusions drawn from a single case require further validation in larger studies, this observation provides valuable clinical insight into therapeutic strategies for critically ill patients with primary plasma cell leukemia.

## Data Availability

The original contributions presented in the study are included in the article/supplementary material. Further inquiries can be directed to the corresponding author.

## References

[1] Bisht K Fukao T Chiron M Richardson P Atanackovic D Chini E . Immunomodulatory properties of CD38 antibodies and their effect on anticancer efficacy in multiple myeloma. Cancer Med. (2023) 12:20332–52. doi: 10.1002/cam4.6619. PMID: 37840445 PMC10652336

[2] Bisht K Walker B Kumar SK Spicka I Moreau P Martin T . Chromosomal 1q21 abnormalities in multiple myeloma: a review of translational, clinical research, and therapeutic strategies. Expert Rev Hematol. (2021) 14:1099–114. doi: 10.1080/17474086.2021.1983427. PMID: 34551651

[3] Dou X Liu R Liu Y Peng N Wen L Wu Y . Efficacy and safety of first-line treatment with anti-CD38 monoclonal antibody-based regimen for primary plasma cell leukemia. Natl Med J China. (2024) 104:499–506. doi: 10.3760/cma.j.cn112137-20231005-00634. PMID: 38317361

[4] Facon T Moreau P Špicka I Suzuki K Yong K Mikhael J . Isatuximab in combination with carfilzomib and dexamethasone in 1q21+ patients with relapsed/refractory multiple myeloma: long-term outcomes in the phase 3 IKEMA study. Hematological Oncol. (2024) 42:e3258. doi: 10.1002/hon.3258. PMID: 38402467

[5] Favas Karimbanathottathil M Yoosuf BT Mamatha M Bansal D . Comprehensive safety evaluation of isatuximab in multiple myeloma using disproportionality analysis of FAERS and meta-analysis of randomized controlled trials. Sci Rep. (2024) 14:31859. doi: 10.1038/s41598-024-83014-1. PMID: 39738354 PMC11685498

[6] Hicks LK Messersmith HJ al Hadidi S Banerjee R Derman BA Kumar S . Treatment of multiple myeloma: ASCO–Ontario Health (Cancer Care Ontario) living guideline. J Clin Oncol. (2026) 44:914–41. doi: 10.1200/jco-25-02587. PMID: 41494138

[7] Martin TG Corzo K Chiron M van de Velde H Abbadessa G Campana F . Therapeutic opportunities with pharmacological inhibition of CD38 with isatuximab. Cells. (2019) 8:1522. doi: 10.3390/cells8121522. PMID: 31779273 PMC6953105

[8] Martino EA Derudas D Rossi E Stefanoni P Mangiacavalli S Zamagni E . Efficacy and prognostic indicators of isatuximab, pomalidomide, and dexamethasone (IsaPd) in daratumumab-refractory multiple myeloma patients: a multicenter real-world study. Hematological Oncol. (2025) 43:e70042. doi: 10.1002/hon.70042. PMID: 39898517 PMC11789454

[9] Musto P Engelhardt M van de Donk NWCJ Gay F Terpos E Einsele H . European Myeloma Network Group review and consensus statement on primary plasma cell leukemia. Ann Oncol. (2025) 36:361–74. doi: 10.1016/j.annonc.2025.01.022. PMID: 39924085

[10] Redondo-Muñoz J García-Pardo A Teixidó J . Molecular players in hematologic tumor cell trafficking. Front Immunol. (2019) 10:156. doi: 10.3389/fimmu.2019.00156. PMID: 30787933 PMC6372527

[11] Schinke C Boyle EM Ashby C Wang Y Lyzogubov V Wardell C . Genomic analysis of primary plasma cell leukemia reveals complex structural alterations and high-risk mutational patterns. Blood Cancer J. (2020) 10:70. doi: 10.1038/s41408-020-0336-z. PMID: 32555163 PMC7303180

